# The paediatric flat foot proforma (p-FFP): improved and abridged following a reproducibility study

**DOI:** 10.1186/1757-1146-2-25

**Published:** 2009-08-19

**Authors:** Angela Margaret Evans, Hollie Nicholson, Noami Zakarias

**Affiliations:** 1 School of Health Science, Division of Health Science, University of South Australia, City, East Campus, North Terrace, Adelaide 5000, South Australia, Australia; 2 Country Health SA: Yorke and Lower North Health Service, Clare, South Australia, Australia; 3 Port Pirie Regional Health Service, Country Health SA, Port Pirie, South Australia, Australia

## Abstract

**Background:**

Concern about a child's flat foot posture is a common reason for frequent clinical consultations for an array of health care and medical professionals. The recently developed paediatric flatfoot clinical-care pathway (FFP) has provided an evidence based approach to diagnosis and management. The intra and inter-rater/measurer reliability of the FFP has been investigated in this study.

**Methods:**

From a study population of 140 children aged seven to 10 years, a sample with flat feet was identified by screening with the Foot posture index (FPI-6). Subjects who scored ≥ 6 on the FPI-6 for both feet became the study's flat foot sample. A same subject, repeated measure research design was used for this study which examined the reliability of the FFP in 31 children aged seven to 10 years, as rated by three examiners.

**Results:**

Approximately half of the items of the FFP showed less-than-desirable inter-rater reliability, arbitrarily set at the conventional 0.7 level (intra-class correlations). Removal of the unreliable items has produced a shorter; more relevant instrument designated the paediatric flat foot proforma (p-FFP).

**Conclusion:**

The p-FFP is a reliable instrument for the assessment and resulting treatment actions for children with flat feet. Findings indicate that the simplified p-FFP is a reproducible instrument for the clinical assessment of flat foot in mid-childhood.

## Background

The significance of "flat feet" continues to debated within the general community, medical and allied health fields, as it has for decades [1-12]. Although flat foot in childhood is a common diagnosis and well established clinical term, there is a lack of a reliable and reproducible tool for the assessment of this condition.

The paediatric flat foot is a controversial topic. Whilst many reports relating to flat feet/pes planus/pes valgus have occupied the medical literature[4,6-8,10,11,13-20], there remains a paucity of well-founded, scientific knowledge about this common condition. A definitive definition is lacking and children's flat feet continue to be diagnosed by a plethora of methods, from observation to clinical measurements and imaging, which are largely unsubstantiated in terms of the reliability and validity of the same. Flat feet, as a postural morphology, have long been associated with pain and disability (eg an exclusion from military service in both World Wars) and thus are often a concern to parents from a preventative perspective of their children's health and mobility.

The reported prevalence of paediatric flat foot varies in the literature and ranges from approximately 3 – 15% [1,5,21]. Views of treatment are contentious [4,6,22] and there is little longitudinal data to provide evidence of the efficacy of different regimen [23-25].

**Figure 1:**
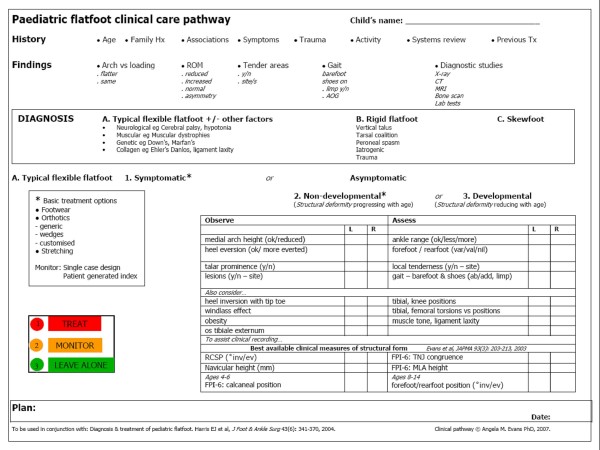
**The paediatric flatfoot clinical-care pathway (FFP), as used in this reliability study**.

Clinicians often disagree about the management of flatfeet [26,27], partly because there is no standard approach to assessment or classification of flat foot sub types (eg rigid, flexible, symptomatic, developmental). The flat foot clinical pathway or proforma (FFP) developed in previous work [28], offers an evidence based clinical tool for the evaluation of this common childhood condition (Figure 1).

The FFP offers a structured checklist approach to significant clinical findings viz. arch shape (weight bearing compared to non-weight bearing), range of motion (reduced, increased or asymmetry), tender areas (joint location and presentation eg swelling), gait (limp, asymmetry, or altered angle of gait; examined shod and barefoot), and diagnostic studies (as available, required).

The focus of the FFP is centred on an accurate diagnosis of the suggested sub-types of paediatric flat foot (flexible, rigid, skewfoot, other). For the purposes of this study the FFP observations/assessments items were collected in categorical form eg medial longitudinal arch: ok/reduced, heel inversion with tip toe: yes/no, tibial, knee positions: medial/straight/lateral. Clinical measures were collected in the units of measure (degrees, millimetres) or as scaled observations for FPI-6 criteria. The FPI-6 was used as described elsewhere [29] to assess all subjects overall foot posture. The FPI-6 consists of six separate scaled observations (-2 to +2) which are then summed to give a total score/foot. The FPI-6 total scores range from -12 (highly supinated) to +12 (highly pronated) and provides a scaled rating of static foot posture.

The present study was undertaken to assess the reproducibility of the FFP, when used by the same observer and between different observers evaluating the same subject.

Ethical approval was obtained from the Human Research and Ethics committee at the University of South Australia. Two primary schools in Port Pirie were approached and consented to being involved in the study. Consent forms were returned from the parents of 140 children, aged between seven and 10 years.

## Methods

All 140 paediatric subjects were initially assessed by the one examiner (AE) using the Foot Posture Index (FPI-6) to establish designated flatfoot status [29-31]. Of these, 31 subjects returned an FPI-6 score of ≥ = 6 for both feet, indicating bilateral flat foot [29] and these subjects were selected for the flatfoot proforma reproducibility study.

All measures were recorded against each child's allocated identity (ID) code. Coloured paper (eg pink, green, blue) to designate each investigator, were used for the FFP charts, with 'am' on the first examination session sheets and 'pm' on the repeated examination sheets. All measures were performed with children dressed but with shoes and socks removed.

The reliability study followed standard protocol as a same subject, repeated measures investigation by three examiners. Each child removed their shoes and socks and stood on a low table approximately 0.5 m in height. The child was asked to look straight ahead (out of a window) whilst their feet were examined. Each of the investigators observed the child's feet and recorded their findings via the FFP. Each child's gait was also briefly observed by each investigator. The total foot examination time took approximately five to10 minutes for each child for each investigator.

The second examination session took place at least three hours after the first session. The second examination session was identical to the first, excepting the collection of anthropometric data which was only collected at the initial examination. At the completion of this examination shoes and socks were replaced, and the children were returned to their classrooms.

All examination findings were entered into a database for statistical analyses of the investigator's examining reliability (both intra and inter-rater) utilizing the FFP. To preserve confidentiality, only the children's ID codes were entered with this data.

### Data analysis

Data were entered and all analyses were performed using constructed data sets in SPSS version 15 (SPSS Science, Chicago, Illinois) and Microsoft Excel 2000 (Microsoft Inc, Redmond, Washington) software packages.

To determine intra-rater agreement, intraclass correlation coefficients (ICCs) were calculated (model [3,1] based on two-way analysis of variance, mixed effect with consistency). 95% confidence intervals were also calculated for each rater's measures [32].

To determine inter-rater agreement, the intraclass correlation coefficient was used in its most conservative form (model [1,1] based on two-way analysis of variance, random effect with absolute agreement) were calculated, within 95% confidence intervals.

The ICC, widely used for reliability analyses, reflects both correlation and agreement and provides a single index among two or more ratings, which was a requirement of this study [33]. Calculating ICCs also made the results comparable with previous studies [34,35].

An acceptable level of reliability was defined, acknowledging that such limits are essentially arbitrary. However, such definitions provide useful "benchmarks" for discussion. Intraclass correlation coefficient values greater than 0.70 indicated good reliability [32] and were used to determine which FFP items might be retained or discarded. Confidence Intervals (95%) were also calculated to show the range of reliability results.

## Results

**Figure 2:**
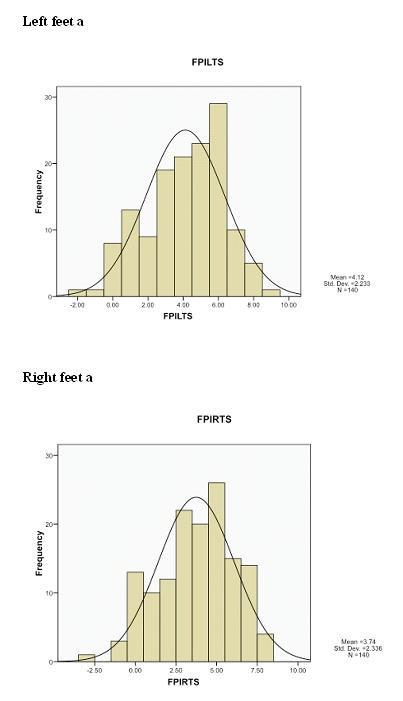
**Foot posture histograms**. The FPI-6 total scores for both left and right feet of the study population (N = 140), children aged seven to 10 years. For both feet the total FPI-6 scores approximated 4, indicative of a 'pronated' status as a regular finding for foot posture in this age cohort.

The foot posture histograms for the study population (N = 140) (Figure 2) showed normal distribution for both left and right FPI-6 total scores. The FPI-6 left foot total score averaged 4.12 (± 2.2) and the FPI-6 right foot total score averaged 3.74 (± 2.3). The FPI-6 scores ranged from -3 to +9 indicating that a range of foot types, ie supinated to pronated, were encountered within this study group, which is important for the external validity of these findings.

The results of the FFP items which returned inter-rater reliability results (ICC [1,1]) of approximately ≥ 0.70 in the flatfoot group (n = 31), are shown in Table 1 (within 95% CI).

**Table 1:** Flat foot group (n = 31, 3 raters) inter-rater reliability analyses.

p-FFP	*ICC (95% CI) – approximated at > 0.7*	
**FINDINGS**		
Tender areas	0.85 (0.75–0.92)	
Gait	0.78 (0.62–0.88)	

**DIAGNOSIS**		
Flat foot type	0.67 (0.46–0.82)	

**OBSERVED**	*Left*	*right*
Medial longitudinal arch	0.78 (0.61–0.88)	0.85 (0.72–0.92)
Heel eversion	0.65 (0.39–0.81)	0.47 (0.42–0.72)
Heel inversion with tip toe	1.00 (1.00)	0.64 (0.27–0.82)
Obesity	0.84 90.74–0.91)	

**ASSESSED**		
Local tender areas	0.84 (0.31–0.87)	0.78 (0.62–0.88)
Tibia, knee position	0.51 (0.19–0.73)	0.67 (0.45–0.82)

**MEASURES**		
RCSP	0.77 (0.62–0.87)	0.19 (-0.35–0.56)
Navicular height	0.66 (0.43–0.81)	0.73 (0.56–0.85)
FPI-6/medial longitudinal arch	0.81 (0.68–0.89)	0.69 (0.47–0.83)

Inter rater results

Intraclass correlations (1,1)

The inter-rater reliability analysis resulted in the items tabulated below being deemed 'acceptable' in terms of the designated reliability cut-off (ICC > 0.70). The mean reliability of the new p-FFP is 0.71 [ICC (1,1)].

Table 2 displays the intra-rater results (ICC [3,1]) for the same FFP items (within 95% CI) as shown in Table 1.

**Table 2:** Flat foot group (n = 31, 3 raters) intra-rater reliability analyses.

p-FFP *ICC (95% CI)*	Rater 1		Rater 2		Rater 3	
**FINDINGS**						
Tender areas	0.61 (0.21 – 0.81)		0.69 (0.36 – 0.85)		0.82 (0.63 – 0.91)	
Gait	0.60 (0.24 – 0.78)		0.81 (0.61 – 0.91)		0.66 (0.31 – 0.84)	

**DIAGNOSIS**						
Flat foot type	0.80 (0.59 – 0.91)		0.34 (-0.35 – 0.68)		0.37 (-0.98 – 0.53)	

	*left*	*right*	*left*	*right*	*left*	*right*

**OBSERVED**						
Medial longitudinal arch	-0.71 (-1.2 – 0.48)	-0.34 (-1.1 – 0.49)	0.64 (0.27 – 0.83)	0.64 (0.27 – 0.82)	1.00	1.00
Heel eversion	0.83 (0.66 – 0.92)	0.61 (0.19 – 0.81)	0.43 (-0.16 – 0.72)	-0.34 (-1.1 – 0.49)	1.00	1.00
Heel inversion with tip toe	-0.34 (-1.1 – 0.49)	0.65 (0.27 – 0.83)	1.00	1.00	1.00	1.00
Obesity	0.92 (0.84 – 0.96)	-	1.00	-	0.53 (0.42 – 0.77)	-

**ASSESSED**						
Local tender areas	0.53 (0.42 – 0.77)	0.27 (-0.51 – 0.65)	0.73 (0.43 – 0.87)	0.67 (0.35 – 0.85)	0.82 (0.63 – 0.91)	0.81 (0.60 – 0.91)
Tibia, knee position	0.38 (-0.27 – 0.70)	0.50 (-0.32 – 0.76)	0.39 (-0.25 – 0.7)	-0.11 (-1.3 – 0.46)	-0.59 (-1.2 – 0.49)	0.81 (0.60 – 0.91)

**MEASURES**						
RCSP	0.79 (0.56 – 0.90)	0.75 (0.48 – 0.88)	0.64 (0.26 – 0.82)	0.12 (-0.81 – 0.57)	0.55 (0.07 – .078)	0.46 (-0.10 – 0.74)
Navicular height	0.82 (0.63 – 0.91)	0.78 (0.54 – 0.89)	0.21 (-0.63 – 0.61)	0.39 (-0.25 – 0.71)	0.89 (0.79 – 0.95)	0.89 (0.76 – 0.95)
FPI-6/medial longitudinal arch	0.88 (0.75 – 0.94)	-0.34 (-1.1 – 0.49)	0.91 (0.81 – 0.95)	0.87 (0.74 – 0.94)	0.51 (-0.01 – 0.76)	0.78 (0.55 – 0.89)

Intra-rater results [n = 31]

Intraclass correlations (3,1)]

Rater 1 was the more experienced clinician, which may be reflected in the intra-rater results. Rater 3 recorded same values in the 'observed' section, limiting interpretation.

**Figure 3:**
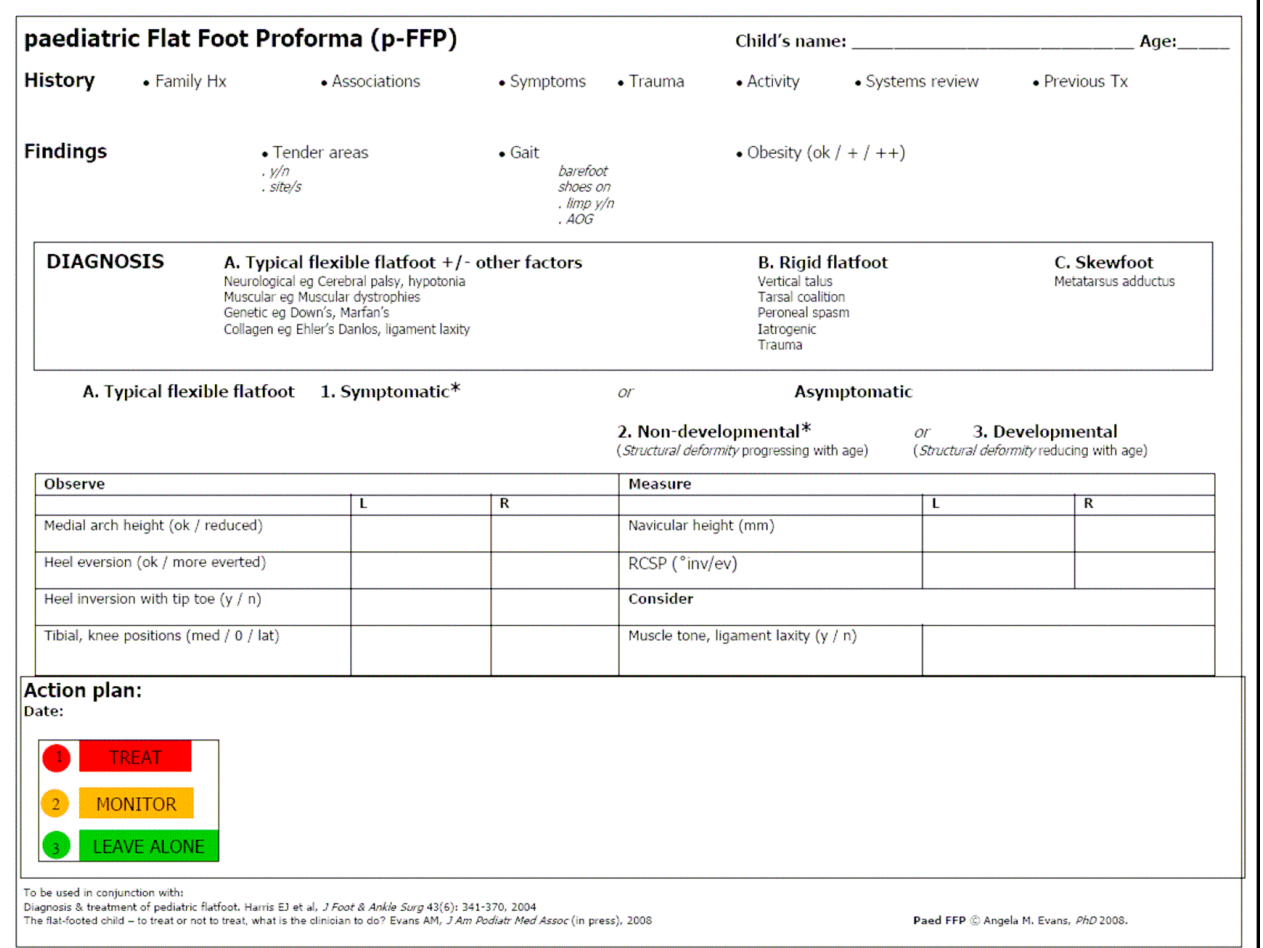
**The paediatric flat foot proforma (p-FFP)**. The new p-FFP has an item reliability mean of 0.71 (ICC 1,1). Treatment is directed for the typical flexible flat foot according to sub-type assessment ie type A1, symptomatic/'red light', treat; type A2, asymptomatic-non-developmental/'orange light', monitor; type A3, asymptomatic-developmental/'green light', leave alone.

Table 3 shows the items/examination area of the FFP and the same when inter-rater reliability (ICC [1,1] ≥ 0.70) levels were applied. The net result of reliability testing effectively halved the number of items remaining (to form the new p-FFP, Figure 3).

**Table 3:** The p-FFP has 29 less items than the original version of the paediatric flat foot instrument (FFP) as a result of the reliability analysis.

	Paediatric flatfoot clinical pathway FFP, 2008	Paediatric flatfoot proforma p-FFP, 2009
History	8	7
Findings	5	3
Diagnosis	3	3
Observations/assessment	40	14
Action	1	1

**Total items**	**57**	**28**

## Discussion

Assessment of the reliability of the individual items of the FFP revealed that many of the items returned poor reliability. As measures, items with poor inter-rater reliability are of little value for clinicians and once identified, usefully discarded. The net effect of this study has been to revise the FFP [28] by eliminating the items with demonstrably poor inter-rater reliability, which by convention were those with ICC of < 0.70 [32]. Approximately half the items failed to meet the set inter-rater reliability standard (ICC > 0.70). The resulting flat foot assessment tool is greater in brevity, reliability and hence general clinical value.

A limitation of this study is that the overall reproducibility of the newly modified p-FFP was not confirmed as part of this study. This issue is being addressed in a project currently underway, but remains a limitation of the present study. The selection of subjects aged seven to 10 years may limit the application of the p-FFP. However, in clinical practice this is a common age of presentation, parental concern and clinical quandary regarding management [36].

The paediatric flat foot proforma (p-FFP) provides a pragmatic standard by which paediatric flat feet can be assessed and management broadly directed. The p-FFP is a compilation of best available evidence [24], consensus guidelines [37] and tested clinical foot posture measures [34]. Within a framework of context (history and signs), the p-FFP is diagnostically rich, yet simple. As a tool, it allows reliable comparison from baselines and between clinicians or researchers. In addition, the p-FFP maintains the simple 'traffic light" framework, making it easy to explain to parents and other medical or health professionals, and ensuring that all are literally on the same evidence-based page when considering the child's flat foot.

It is interesting to note that the focal points of the p-FFP are the presence/absence of symptoms, the arch morphology, and the heel position – all of which, mooted for many years [3,4,6,19,38-40], are now substantiated. The p-FFP directs action largely dependent upon symptoms (treatment indicated, the 'red light'), age (developmental flat foot is normal physiology, the 'green light') and clinical experience (monitoring and simple management of the 'amber light'). Whilst concerning for some [26], this approach is judicious, evidence-based and contemporary [27].

It must be stressed that any clinical pathway, no matter how rigorously evaluated, should always be used in conjunction with good clinical judgement.

## Conclusion

The findings of this study suggest that the modified p-FFP is a more reproducible and reliable tool for the assessment of flat foot in children, than the previously developed version: the paediatric flat foot clinical pathway (FFP). The modified tool, which requires approximately half the number of items is both simpler and less time consuming to use and most importantly demonstrated satisfactory inter-rater/measurer reliability. Within the limitations of the study, these findings support the use of the p-FFP as a clinical tool for the assessment and evaluation of this common childhood condition. However it is recommended that if this instrument were to be used in future research studies of flat foot in childhood, the intra-rater and or if appropriate inter-rater reproducibility of the tool should be tested and recorded prior to data collection to demonstrate and ensure scientific rigour.

## Competing interests

The authors declare that they have no competing interests.

## Authors' contributions

AE conceived and lead the study, participated in data collection, performed the statistical analysis and drafted the manuscript. HN and NZ participated in data collection. All authors participated in protocol development and read and approved the manuscript.
